# Co-Design of an Escape Room for e-Mental Health Training of Mental Health Care Professionals: Research Through Design Study

**DOI:** 10.2196/58650

**Published:** 2025-01-07

**Authors:** Joyce J P A Bierbooms, Wouter R J W Sluis-Thiescheffer, Milou Anne Feijt, Inge M B Bongers

**Affiliations:** 1 Tilburg University Tilburg Netherlands; 2 HAN University of Applied Sciences Arnhem and Nijmegen Netherlands; 3 Eindhoven University of Technology Eindhoven Netherlands

**Keywords:** serious gaming, mental health care professionals, e-mental health, skill enhancement, training

## Abstract

**Background:**

Many efforts to increase the uptake of e-mental health (eMH) have failed due to a lack of knowledge and skills, particularly among professionals. To train health care professionals in technology, serious gaming concepts such as educational escape rooms are increasingly used, which could also possibly be used in mental health care. However, such serious-game concepts are scarcely available for eMH training for mental health care professionals.

**Objective:**

This study aims to co-design an escape room for training mental health care professionals’ eMH skills and test the escape room’s usability by exploring their experiences with this concept as a training method.

**Methods:**

This project used a research through design approach with 3 design stages. In the first stage, the purpose, expectations, and storylines for the escape room were formulated in 2 co-design sessions with mental health care professionals, game designers, innovation staff, and researchers. In the second stage, the results were translated into the first escape room, which was tested in 3 sessions, including one web version of the escape room. In the third stage, the escape room was tested with mental health care professionals outside the co-design team. First, 2 test sessions took place, followed by 3 field study sessions. In the field study sessions, a questionnaire was used in combination with focus groups to assess the usability of the escape room for eMH training in practice.

**Results:**

An escape room prototype was iteratively developed and tested by the co-design team, which delivered multiple suggestions for adaptations that were assimilated in each next version of the prototype. The field study showed that the escape room creates a positive mindset toward eMH. The suitability of the escape room to explore the possibilities of eMH was rated 4.7 out of 5 by the professionals who participated in the field study. In addition, it was found to be fun and educational at the same time, scoring 4.7 (SD 0.68) on a 5-point scale. Attention should be paid to the game’s complexity, credibility, and flexibility. This is important for the usefulness of the escape room in clinical practice, which was rated an average of 3.8 (SD 0.77) on a 5-point scale. Finally, implementation challenges should be addressed, including organizational policy and stimulation of eMH training.

**Conclusions:**

We can conclude that the perceived usability of an escape room for training mental health care professionals in eMH skills is promising. However, it requires additional effort to transfer the learnings into mental health care professionals’ clinical practice. A straightforward implementation plan and testing the effectiveness of an escape room on skill enhancement in mental health care professionals are essential next steps to reach sustainable goals.

## Introduction

### Background

Despite a growing body of evidence for the effectiveness and benefits of e-mental health (eMH), many studies report a relatively low use of eMH in practice [1-3]. eMH means mental health services delivered or supported by digital technologies, such as video calling, self-management and self-tracking apps, web-based treatment modules, and virtual reality (VR) [4,5]. An important factor hampering the use of eMH is its relatively slow adoption by mental health care professionals [5-7]. To a large extent, this slow adoption results from a lack of knowledge and skills regarding eMH among mental health care professionals [7-9].

This lack of skills entails general digital skills but, more importantly, the ability to use an adequate communication approach compensating for a potential lack of nonverbal cues and contextual information. In addition, skills are needed to guide a professional in handling boundaries (eg, expectations regarding reaction time and attainability), choosing appropriate communication channels for each situation, and up-to-date knowledge of the availability of technological possibilities to enrich treatment in mental health care [10-12]. The COVID-19 pandemic caused a significant increase in video calling and, subsequently, the skills to apply such technologies for therapeutic purposes. However, mental health care professionals are still hesitant to pursue broader incorporation of eMH (ie, using different eMH tools in a context where remote treatment is a choice rather than an imposition) into their therapeutic practices because of the skills required [13]. Thus, to increase the adoption of a broad range of eMH tools, there is a persistent need to enhance mental health care professionals’ skills in using eMH.

Many training options have recently been developed to capacitate mental health care professionals in using eMH [14,15]. The introduction and development of web-based treatment platforms have brought different programs to acquire the technical skills to use such a platform. Examples are applying web modules, written client interactions, and video sessions instead of real-life therapeutic interactions. Besides the classical training approach, there are several eLearning options to support mental health care professionals in digital skill development [16]. However, research on the effectiveness of different learning and training strategies in health care has shown that traditional forms, such as classical instructions and eLearning, generate insufficient learning outcomes [17,18]. This can be explained by a lack of experiential learning in conventional learning methods; there is a need for a “learning by doing” approach. The learning by doing approach is more engaging, more realistic, and contributes to a larger self-efficacy [17,18]. Thus, there is a need to pursue new, more innovative, and experiential solutions to train mental health care professionals in using eMH.

To gain a feeling of self-efficacy regarding the application of eMH, a potential strategy is to offer mental health care professionals training possibilities based on the concept of serious gaming [19]. Serious games are physical or digital games applied to learn or acquire skills and usually offer a combination of educational content and engagement [19,20]. Escape rooms have emerged as a popular instructional approach within serious gaming, attracting growing interest from researchers and educators [21]. An escape room is a game in which participants are confronted with a storyline, usually locked into a room, and need to solve puzzles, search for information, and follow leads to free themselves from the room within a limited amount of time. Escape room games are increasingly used for learning [22-24].

The benefit of an escape room may be that it offers the option to train skills by gaining hands-on experiences (experiential learning). Escape rooms create realistic learning environments that foster meaningful participation and provide a supportive atmosphere for experimentation [25]. In contrast, other methods (eg, classical instructions or eLearning) mainly focus on providing instructions on using an artifact or service [18,26]. In addition, an escape room is focused on the learning process while simultaneously being educational and fun, which makes it much more engaging to participate in training [27,28].

The underlying learning mechanisms of an educational escape room (EER) are often described in terms of the self-determination theory (SDT) [29,30] and the flow theory [30,31]. The SDT refers to fundamental psychological needs such as autonomy, competence, and relatedness to others [29,30]. EERs provide a safe environment that can be tuned to the player’s competencies, and an appropriate storyline helps players relate the competencies demanded in the EER puzzles to competencies required in real-world situations. Together, they increase a player’s sense of autonomy, and they can create a positive experience in self-determination. The flow theory explains the occurrence of someone’s complete immersion into a specific task based on positive experiences [30,31]. This “flow” can be achieved in serious gaming if the challenges meet the player’s knowledge and abilities. By attempting to address these fundamental needs in a playful environment, users can have a positive learning experience [32].

Theoretically, these mechanisms provide a solid argument for the use of EERs to train mental health care professionals’ skills in successfully integrating eMH into their therapeutic practice [22,23]. However, as Vorderobermeier et al [30] indicate, the success of EERs is hardly ever tracked back to these mechanisms. To contribute to the emerging field of research into EERs, we include an evaluation of the participants’ learning experience in relation to these learning mechanisms.

### EERs in eMH

In eMH, the concept of EERs is recognized as a safe and interactive environment to explore eMH in different possible therapeutic situations [30,32] while providing a safe and social environment for training in mental health care. EERs provide an opportunity to experiment with otherwise complex or expensive therapeutic situations [33,34]. As EERs are an emerging phenomenon, there are also some recent examples of research into EERs in the context of eMH.

In the context of raising awareness of severe mental illnesses (SMIs), Rodriguez-Ferrer et al [35] studied the effectiveness of an EER in educating first-year nursing students (a nonspecialized audience) in experiencing the stigmas associated with SMI. The EER was developed with professionals in SMI and the effect of raising awareness was tested using a randomized controlled trial experiment. Apart from the primary objective (raising awareness of SMI), the EER objectives were to determine whether an entirely web-based version was effective and whether participants were sufficiently immersed in the experience. The EER in this study is different in the sense that it is aimed at experiential learning, involving both digital and physical interactions. Moreover, the EER developed in this study is aimed at a professional audience, which means that the challenges are tuned toward expert knowledge and an in-depth skillset rooted in ample experience in treating mental illnesses [35].

Aragon [36] designed and studied the use of an EER for mental health nursing students attending an accelerated associate degree program. The topic of learning was communication and collaboration strategies in mental health. The design of the EER took a pragmatic, digital form to simplify the strategy for the development, distribution, and deployment of the EER in the educational program. The authors studied the potential learning effect of the digital EER but found no significant difference compared to students who were not exposed to the EER. The EER in this paper is different, similar to the EER studied by Rodriguez-Ferrer et al [35] mentioned earlier, as it targets a professional audience and aims to provide a more physical, collaborative learning experience. Moreover, the EER by Aragon [36] was solely designed by one researcher, whereas this paper proposes a co-design process to include the views, needs, and goals of the target audience in the design of the EER. Finally, the work by Aragon [36] briefly discusses the underlying mechanisms of the EER, namely (1) the potentially positive effects of collaboratively solving nursing challenges and (2) the strong relatedness of the puzzles with the curriculum thus providing the students with alternative means to consolidate what was learned during the program [36]. The EER in this paper makes a clear choice to define the underlying mechanisms in the SDT theory and the flow theory.

There are more EERs for eMH, such as the one proposed in the study by Petkari and Calvo [37], and the number of EERs is likely to increase over time as its potential is currently widely researched. Similar to the studies discussed earlier, Petkari and Calvo [37] focused on EERs as alternative or additional elements for existing curricula, targeted at soon-to-be professionals. In those contexts, the design of the EER is mostly in the hands of educators or researchers, while the professionals are only involved in evaluations [37].

In a previous paper, we described the careful considerations we took to aim for a successful design and subsequent deployment of an EER that is tailored toward experiential learning about (innovative) digital elements suitable for blended therapies [38]. This paper describes the execution of a preliminary version of the EER.

This study aims to (1) further pursue the ambitions of a co-designed EER for training mental health care professionals in eMH, (2) investigate whether an EER would be an acceptable solution for this purpose, and (3) determine whether the SDT theory and flow theory are recognized as effective mechanics based on the experience of professionals, thus making a contribution to the field of eMH and the emerging field of EERs in the context of health care.

### Co-Designing an EER for eMH Therapy

On the basis of these encouraging arguments and inspiring examples of eMH EERs in pursuing the development of an escape room, we gauged practitioners’ first reactions and expectations regarding this idea. This delivered mostly positive responses: mental health care professionals expect an escape room to be innovative, engaging, inviting, and much more reasonable than the traditional and familiar eMH training methods. Moreover, based on our user requirements analysis [39], we aimed to develop a physical escape room. One argument is that a primary obstacle mental health care professionals reported in the user requirement analysis is that they are unacquainted with the possibilities of eMH. On the basis of this, we assumed that a physical room, with a facilitator present to moderate and explain, if necessary, would yield better results. A physical escape room provides more opportunities for collaboration [40]. Moreover, we learned from the user requirements analysis that users preferred a learning context in which they met others and could discuss with colleagues. Finally, playing an escape room is not a stand-alone solution. It should be part of a holistic experience, including a clear introduction and a reflection afterward, where participants can transfer the value of their eMH experience in the escape room to their real-life work practice [39].

As we aimed to develop a serious game for training mental health care professionals in using eMH, it is important to classify an escape room according to the gameplay/purpose/scope model for serious games [41]. This allows a better understanding of an escape room as a serious game and whether it meets our aim of developing a game with educational content. According to the definition by Djaouti et al [41], which distinguishes game-based from play-based activities by noting that the latter lacks a clear goal, we can, we can classify the gameplay of our intended escape room as game-based*.* The goal of the escape room is to enhance knowledge of the use of eMH by mental health professionals and train them to use eMH tools in different situations. That immediately brings us to the purpose of the escape room, which, in the classification by Djaouti et al [41], would be training. The intention of the escape room is to improve the performance of mental health professionals in using eMH, including how to use certain techniques as well as how to use these techniques in a particular context. We could also argue that there is a data exchange component in our escape room, as we also aim to encourage the users to help each other solve the puzzles and learn from each other’s experiences. The scope of the escape room is health care, with mental health professionals as the main target audience. However, there is also the possibility that an escape room to train eMH skills is incorporated into educational content at vocational or higher professional education [41].

### Objectives

This paper presents the co-design process of developing an escape room for training mental health care professionals in eMH. By assessing the expectations and experiences regarding an EER in mental health care, we aim to evaluate the perceived usability and to gauge the effect of underlying mechanisms rooted in SDT and flow theory for training mental health care professionals’ eMH skills.

## Methods

### Study Design

**Figure 1:**
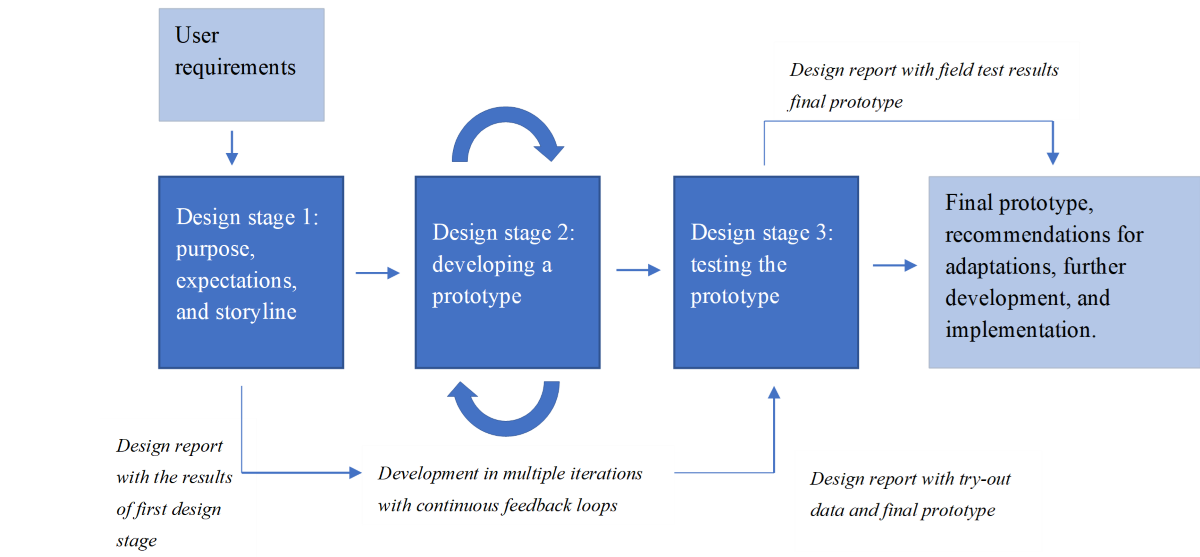
Three design stages in the development of an escape room for e-mental health (eMH) training.

To shape the development and explore the perceived usability of an escape room, we used a process of 3 design stages, in which a testable prototype of an escape room for mental health care professionals was developed. In this “research through design” process, the design of the escape room is part of a research project in which the design activities contribute to generating knowledge [42]. Zimmerman et al [43] define research through design as “a research approach that employs methods and processes from design practice as a legitimate method of inquiry.” This knowledge is applied at each stage to iteratively develop prototypes within the design process [42,43], leading to the final prototype. To explore the potential of an escape room, users’ experiences and ideas are gathered on whether the escape room works as intended and whether it lives up to the promise of a concept for knowledge and skill enhancement. With this research through design approach, we seek to contribute to evidence-based decisions on the implementation, including potential adaptations and strategies to maximize the users’ acceptance of the concept. This means that the design of an escape room is explored for suitability in the practice of mental health care and for its potential to address the aim of enhancing professionals’ skills in using eMH. We organized 9 co-design sessions in 3 design stages (Figure 1). The escape room’s purpose, expectations, and storyline ideas were formulated in the first design stage. These ideas were further translated into a gaming concept (ie, an escape room prototype), assessed for suitability in the second stage. In the third stage, a prototype was tested with end users (ie, mental health care professionals). Data from all stages within this process contributed to exploring the concept’s perceived usability. In the Exploration of an Escape Room in 3 Design Stages subsection in the Results section, we report on the specific procedures and results of the co-design sessions. In doing this, we integrate the procedures and results to describe each design stage as a whole.

### Research Context

The study was conducted at a mental health care organization in Eindhoven (Geestelijke Gezondheidszorg Eindhoven en De Kempen, GGzE), in the southern part of the Netherlands. GGzE delivers specialized mental health care for people with various psychiatric and psychological issues (eg, depression, anxiety disorders, psychosis, autism, and personality disorders). Mental health care professionals working at GGzE include (specialized) psychologists, psychiatrists, nurses, and social workers. GGzE treats approximately 12,000 clients annually and has 2368 employees [44]. The research involved researchers from Tilburg University, Eindhoven Technical University, and Fontys University of Applied Sciences. Through this collaboration between universities, we combined expertise from social science, human and technology interaction, and game design. This combination of expertise vouches for a project in which all necessary aspects of a useful design process (eg, technique, human attitudes and behavior, and serious gaming concepts) and their interaction are considered.

### Participants

#### Stages 1 and 2

The co-design sessions, including 1 web version in stage 2, were held with 5 mental health care professionals (psychologists: n=2, 40%; nurses: n=2, 40%; and social worker: n=1, 20%), 1 innovation staff member, 3 researchers, 3 research laboratory leaders, and 2 game designers. Together, they formed the co-design team for this stage in the design process (Table 1). In line with the existing literature on co-design, we involved stakeholders from the three areas of expertise that were needed for the design project [45-47]: (1) the mental health care professionals that represent the end users of the escape room, the innovation staff that will be involved in launching the escape room in practice; (2) the game designers who have expertise in the development of an escape room; and (3) researchers that are involved in exploring the usability of and experiences with an escape room. The mental health care professionals and the innovation staff members worked at GGzE. They were recruited through an announcement on the organization’s internal communication platform and through network contacts of the project team, that is, a convenience sampling method was used. Mental health care professionals with varying levels of expertise were included in this stage of the development process. After their initial agreement, the participants were further informed about the project, the purpose of their participation, and how their data would be handled.

**Table 1:** Participants’ roles in the development of an escape room for e-mental health (eMH) training.

Participants	Design stage 1	Design stage 2	Web version (design stage 2)	Design stage 3
Psychologists	Participants (n=2)	Participants (n=2)	—^a^	Participant field study session (n=3)
Nurses and therapists	Participant (n=1)	Participants (n=2)	Participant (n=1)	Participant field study session (n=2)
Social workers	Participant (n=1)	—	—	Participant field study session (n=5)
Mental health professionals’ team for web-based treatment	—	—	—	Participant test session 2 (n=4)
Innovation staff members	Participant (n=1)	Participants (n=2)	Participant (n=1)	Participant test session 1 (n=2) Facilitator field study sessions 1 to 3 (n=1)
Researchers	Participants (n=3)	Participant (n=1) Observers (n=2)	Participant (n=1)	Observant field study sessions 1-3 (n=2)
Research laboratory leaders	Facilitator (n=1) Participant (n=1) Observer (n=1)	Observers (n=3)	Participant (n=1)	—
Game designers	Facilitator (n=1) Participant (n=1)	Facilitator (n=1) Observant (n=1)	Facilitator (n=1)	—

^a^Not applicable.

#### Stage 3

In the third stage of the design project, we first asked a purposively sampled selection of mental health care professionals, namely 4 highly experienced eMH therapists from the team for web-based treatment and 2 members of the innovation staff of GGzE, to participate in 2 test sessions. The 2 test sessions secured a sufficient perceived flow of the escape room workshop (ie, including introduction and reflection). They eliminated further technical and practical issues concerning the logistics escape room (Table 1). Subsequently, we invited therapists from all disciplines within ambulatory specialized mental health care to participate in 3 field study sessions to test whether the final prototype of the escape room would be suitable as a concept to enhance mental health care professionals’ skills in using eMH (Table 1). The first session included 2 psychologists, 1 nurse specialist, and 1 social worker. The second session involved 5 therapists engaged in social work and supporting clients’ daily activities. Finally, a clinical psychologist and a verbal therapist tested the escape room in the third session. All mental health care professionals in the 3 field study sessions had some essential experience with eMH (ie, using a web-based module and video calling on a nonfrequent basis).

### Data Collection and Analysis

Detailed notes were taken for all co-design sessions in all stages and were used to compose written reports. These reports were checked with the participants, after which themes were identified to describe, organize, and interpret the data [48]. In addition, video recordings were made of the sessions in stage 3, which were used to verify and complement the written reports. In the first stage, the 2 sessions were analyzed individually because of the difference in nature of the sessions. In the second stage, the written reports were summarized into recommendations for the design team, which they used in the next iteration of the design. In the third stage, the first 2 test sessions were analyzed together, and the 3 field study sessions were also combined for analysis.

We used the Standards for Quality Improvement Reporting Excellence reporting guidelines to check the completeness and improve the quality of our manuscript [49]. A completed checklist can be found in Multimedia Appendix 1.

### Ethical Considerations

This project was approved by the ethics review board of Tilburg University (EC-2013.14) and by the scientific committee of mental health care in Eindhoven (the Netherlands), where the participants were recruited. All participants were informed about the research and allowed to withdraw at any moment. All participants signed the informed consent form. Data in this project were anonymized and cannot be linked back to participants. Participation was voluntary, without financial compensation.

## Results

### Exploration of an Escape Room in 3 Design Stages

We describe the 3 stages of our research through the design process subsequently (Figure 1). As explained in the Methods section, we elaborate on the specific procedures we followed in each design stage to gather the data, the results generated, and how the findings within all 3 stages provide insights into the usability of an escape room as a game-based solution for eMH training for mental health care professionals (Figure 1).

### Design Stage 1: Purpose, Expectations, and Storyline

#### Overview

In the first co-design session of design stage 1, which took place in December 2019, a joint purpose and common ground for the escape room were formulated. Participants were invited to work in subgroups to discuss the following: (1) the current situation, including the mental health care professionals’ attitude regarding eMH; (2) a vision of how future mental health care professionals should be equipped and accommodated to integrate eMH into their daily practice; and (3) the requirements regarding an escape room to transition from the current to the future mental health care professional, in which eMH plays a central role, including the success and failure factors for this concept.

This was done by exchanging ideas, associating concepts, and explaining the relationship between eMH and the work of mental health care professionals.

Following this, a second co-design session was organized, aimed to deliver the storyline for the envisioned escape room. Information was gathered on the particulars of clients in mental health care, the mental health care setting in general, the various treatments and services available, including eMH, and the different client journeys that occur. This provided information on the requirements regarding the content of the game. The designers delineated a fictitious client and a story to form the basis of the escape room game. This initial storyline was verified with the mental health care professionals of the co-design team and was adjusted accordingly.

#### Purpose

In the first co-design session, the participants mentioned that many mental health care professionals are still convinced that the best way to treat a client is through face-to-face interaction. There is still a feeling of eMH being imposed by financial incentives, offering very limited job enrichment for mental health care professionals and very limited options to enrich treatment possibilities for clients. As a result, practitioners often need more interest or time to explore the possibilities of eMH. An important driver for mental health care practitioners to use eMH in the future would be that they see a clear-cut advantage in it for their clients, such as feeling less of a “patient” and gaining self-control and autonomy. This could be established by gaining positive experiences using eMH, for example, in training. Another aspect of the current attitude of many mental health care professionals mentioned in the co-design session was the lack of knowledge and skills to use eMH. Enhancing this knowledge requires time that needs to be facilitated by the organization they work for.

Despite this attitude, mental health care practitioners see the need to integrate eMH into therapy. The future mental health care professional is seen as someone for whom eMH is a natural part of the job. The session revealed that mental health care professionals consider their profession a craft, and some may see innovation as detrimental to this craft. According to the participants in the session, an escape room should demonstrate the opposite: instead of devaluating the craft, eMH has to reinforce it.

#### Expectations

On the basis of the purpose of the escape room, it is essential that sharing positive experiences and building confidence in using eMH are emphasized when designing a prototype. In the co-design session, it was stated that examples of positive experiences with eMH in clinical practice should be visible in the escape room, along with opportunities for professionals to share their positive experiences. Furthermore, the educational content of the escape room should seamlessly fit various real-life scenarios, making a transfer to everyday practice easy. Specific and well-defined learning goals should provide the opportunity to gain confidence in using technology, and the escape room should consist of customizable elements to fit the particular learning needs. Finally, the escape room should be engaging and contain continuous feedback loops, a recognizable storyline, and multiplayer options for a shared learning experience.

In addition to game-specific criteria, conditional requirements, such as perceived ease of use, a robust (technological) system, and perceived usefulness were considered significant. Furthermore, the social influence of colleagues, the perception of available time, endorsement by management, and an excellent incentive to engage in the escape room are issues that need to be considered. Finally, the escape room should be a complete learning experience with a clear introduction and reflection after playing the game. Mental health care professionals must transfer from the learning experience to their therapeutic practice.

#### Storylines

**Figure 2:**
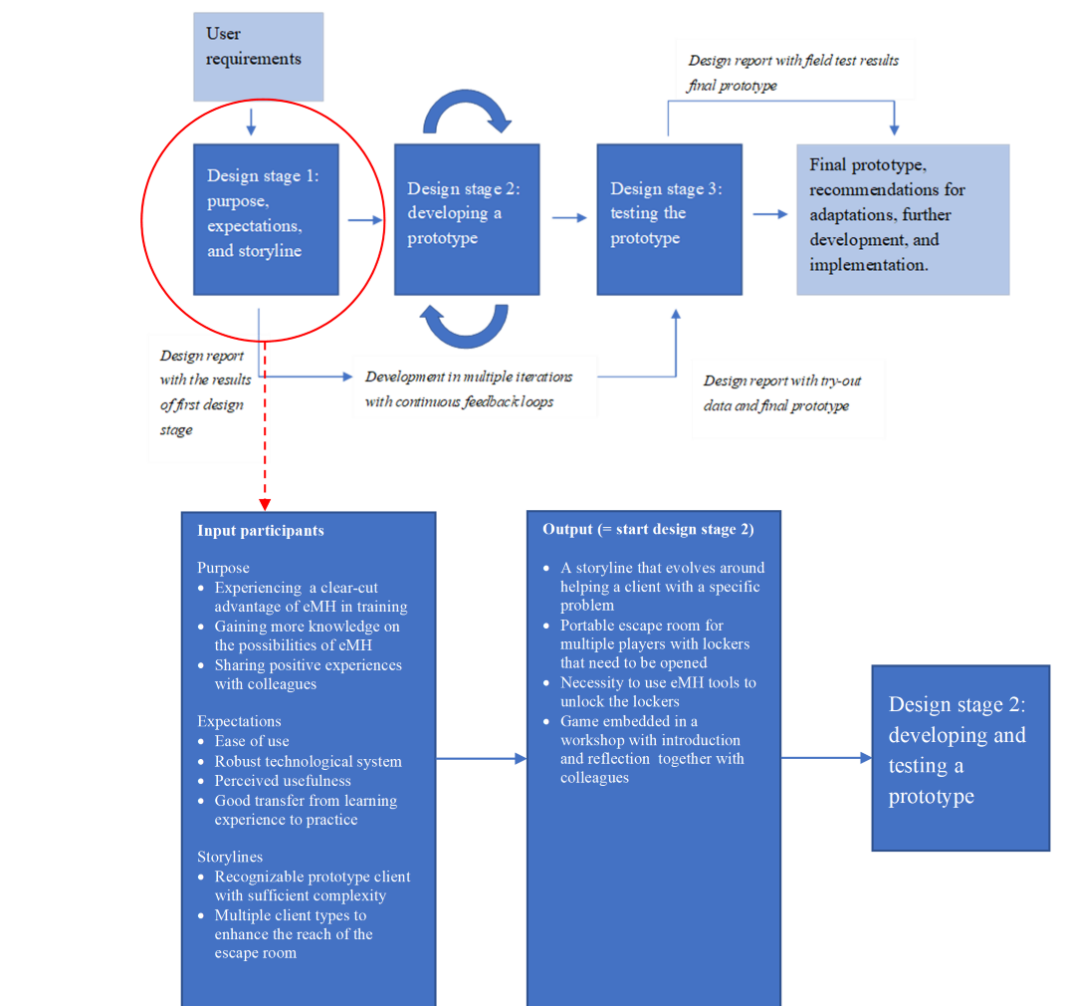
Results of the first design iteration in the development of an escape room for e-mental health (eMH) training.

On the basis of the input in the first session, the designers crafted a storyline for the escape room, which was presented to the co-design team. In their reflection on the storyline, the co-design team remarked that the mental health problems of the fictitious client should be more complex to be eligible for specialized mental health care. It should also contain more details to be sufficiently recognizable for therapists. In this stage of the design process, the co-design team also concluded that it would be valuable if there were sets of multiple storylines revolving around different settings or problems in mental health care to enhance the reach among the potential users of the escape room (Figure 2).

### Design Stage 2: Developing a Prototype

#### Overview

**Figure 3:**
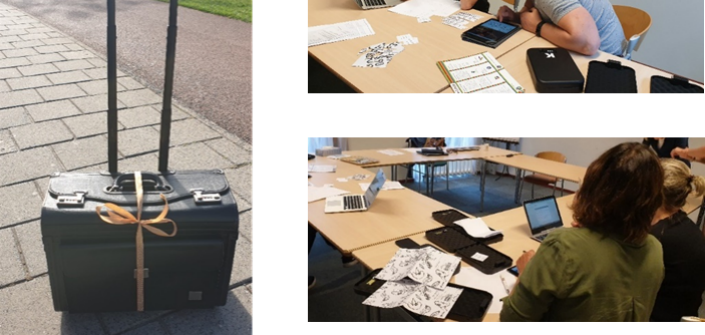
Picture of an escape room setting.

The prototype was presented to the co-design team in February 2020. The prototype of the escape room consists of 2 storylines, which are played separately. In each storyline, the participants are introduced to a client who experiences mental health problems. The challenge in the escape room is to answer questions in the client’s electronic patient file, leading to a final solution to help the client take the next step. These questions can only be answered by finding clues in several locked safes, thoroughly analyzing the information given about the client, and using the eMH tools they see in the room. The eMH tools the participants provided to “help their client” were an electronic diary, a VR application for exposure therapy, biofeedback, a chat function, and web-based treatment platform. All materials used in the escape room are portable, which means playing the escape room is not dependent on a fixed place (Figure 3). The actual game is embedded in a workshop with an introduction to familiarize with the topic, goals, and expectations regarding the escape room and a reflection afterward to discuss the experiences and take-home messages.

In a try-out session, the co-design team tested this prototype by playing the escape room in 3 small groups of 3 participants. This delivered additional data for our research regarding the expectations and experiences of an escape room. At the same time, it provided the designers with feedback for a second prototype. In each round, the participants were given 15 minutes to play the game and solve the puzzles. Each subgroup reflected individually on the prototype directly after playing the escape room. At the end of the session, a plenary discussion took place to summarize the results. The designers developed a second prototype for the escape room based on the feedback. In this phase, a second storyline was also developed. Different eMH tools were added and various connections were made between these eMH tools, the storyline, and the puzzles.

In March 2020, the world was confronted with an unprecedented global pandemic that caused a significant disruption of life as we knew it. Burdened by the consequences of the pandemic and the measures that national governments took to control the virus (eg, social distancing, lockdown, and quarantine), the activities in our design process were also heavily disturbed. Switching to this new situation, we sought to continue the design process by developing a web-based alternative to test the second prototype of the escape room in April 2020. This web version was a digital copy of the physical room, containing the same elements, puzzles, and eMH tools, except for the VR goggles. This second prototype included 2 storylines, each with a fictitious client, and was tested by the co-design team members. One storyline was tested while being moderated by the designers, the other storyline was tested by each member individually, without being moderated.

#### Try-Out Sessions

The feedback gathered during the try-out sessions delivered several expectations and experiences of mental health care professionals regarding an escape room. First, storylines in an escape room benefit from a good match with a client’s pathway in mental health care and reflect truly relatable situations. Moreover, it is essential to focus on mental health problems as an integrated part of everyday life situations. This is important because otherwise, the suggested eMH tools cannot be appropriately linked, and a genuine eMH experience will be missed. This suggestion was also given in response to the final storyline in the previous phase of the design process. Second, there should not be too much focus on solving the puzzles in the escape room, as it diminishes the awareness of eMH in the game. More importantly, the eMH tools should be linked explicitly to the puzzles. The participants should experience a (positive) consequence when choosing, for example, finding the next clue when trying a specific eMH tool or experiencing that someone (ie, the fictitious client) benefits from you as a practitioner choosing to use eMH. Third, variation and differentiation in an escape room vouches for a stronger and fuller user experience. This could be established by multiple storylines (as was stated in the previous phase of the design process), different sets of eMH tools that can be used, and variations in the level of difficulty in the escape room. In this way, the escape room can be played multiple times and addresses different levels of expertise, either regarding eMH or playing an escape room. Fourth, there should be a clear conclusion to the escape room and, consequently, a euphoric feeling when finishing the game. To this end, the purpose at the beginning of the game may need to be more apparent. Fifth, it should be beyond any doubt that the experience of eMH in the escape room can also be transferred to the daily practice of a mental health care professional. Otherwise, the escape room will not be considered credible.

#### Web Version

From the tryout, it became clear that the experience of a web version of an escape room does not closely resemble the experience of an escape room in real life. Due to several technical hassles, the game’s flow was hindered multiple times. Most importantly, it impeded the possibility of team interactivity, working together, and exchanging ideas about eMH, which was mentioned multiple times in the design process as one of the critical success factors for an escape room.

#### Final Prototype

After receiving the feedback on the web version of the escape room, the designers continued developing a prototype that was fit for testing by end users. The co-design team subsequently assessed a final prototype in July 2020. After minor adaptations, this prototype was finalized in September 2020. Following this, a field study was planned (stage 3) to gather the user experiences of the escape room among the end users, that is, mental health care professionals from various disciplines (Multimedia Appendix 2).

### Design Stage 3: Testing the Final Prototype

#### Test Sessions

In 2 test sessions (September and October 2020), we first checked the flow of the escape room workshop and the functioning of the materials and technology. This delivered a positive outcome regarding the potential of the concept for exploring eMH and, in that way, enhancing mental health care professionals’ knowledge about the possibilities of technology in therapy. The participants of these sessions shared several thoughts on how to improve the workshop before starting the field study sessions with end users.

The first finding in the test sessions was the importance of good functioning of the content and technical aspects of the escape room to ensure a good flow in the game. Besides a good flow in the game, a facilitator should lead a good introduction and reflection to complement the learning experience.

According to the participants in the test sessions, a good explanation of the purpose and relevance of the escape room and presenting a game manual to maximize the effect of playing the escape room is necessary. One participant particularly mentioned the importance of stressing the educational purpose and preventing the feeling that the workshop is merely something for fun. After playing the escape room, reflecting on the purpose is important. According to the participants, there should be a clear take-home message, discussion on the experiences gained in the escape room, and acquired knowledge to share with colleagues afterward.

Another issue raised during the 2 test sessions was the need to continuously adapt the storylines or create the flexibility to insert additional storylines in the escape room game. Two storylines may be too few to address all therapeutic contexts, all levels of complexity in mental health care, and all eMH possibilities that could add value. The participants also marked a sense of reality in the storylines. Opportunism should be avoided because it should not imply that technology will always vouch for a positive treatment outcome. For example, in VR, the escape room should not pretend that it is something that instantly makes everything better. This was regarded as an essential issue to warrant the credibility of the escape room. However, the same session also mentioned that experiencing eMH tools in this manner created awareness about escape room’s potential. At the same time, the biofeedback application also showed that it is possible to visually track panic attacks in such an application. The final aspect regarding the mentioned storylines is using the correct professional jargon in the escape room. At one point, a participant stated that the way things were written in the escape room did not reflect the language a therapist would use. In contrast, language and jargon appeared very personal, making it difficult to always be accurate in an escape room. This emphasizes the fact that there is a large diversity among mental health care professionals and the clients they are treating.

Finally, the participants discussed the complexity of the game itself. Some participants found figuring out the puzzles and clues challenging, but others remarked it was too simple. Some solutions were easily found without using the eMH tools in the escape room, resulting in an ineffective learning experience. Some participants in the test sessions suggested creating multiple levels of complexity in the escape room to address several types of game players (ie, level of experience with escape room games) and other kinds of therapists.

**Figure 4:**
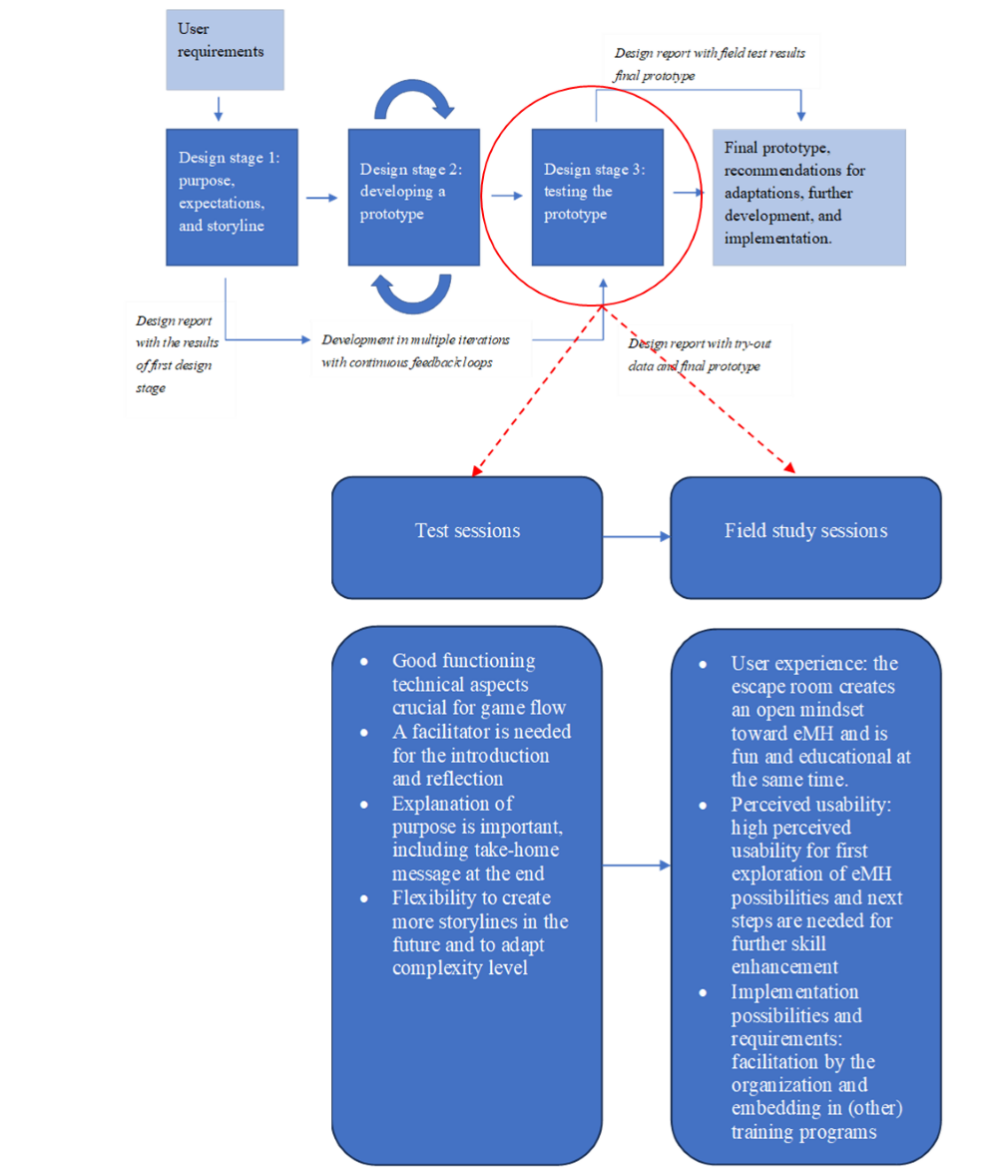
Results of the test and field study sessions (third iteration) in the development of an escape room for e-mental health (eMH) training.

On the basis of the results of these 2 test sessions, minor adjustments were made regarding the complexity of the puzzles to ensure that clues and solutions could not be found without using eMH tools. Furthermore, the introduction and reflection were refined to embed the escape room game in a workshop format (Figure 4).

#### Field Study Sessions

##### Overview

Hereafter, the workshop was tested in 3 sessions with mental health care professionals from different areas of expertise. A total of 11 mental health care professionals participated in these field study sessions (November and December 2020 and June 2021) and were asked to fill in a questionnaire (Multimedia Appendix 3) at the end of the workshop. Their answers formed the basis for a discussion about their user experiences. The questionnaire consisted of an overall experience rating (question 1), three parts to reflect on (1) the quality of the introduction (questions 2-4), (2) the game itself (questions 5-11), and (3) the reflection (questions 12-15), overall questions regarding the perceived usability (questions 16-19), and a question on strengths and suggestions (question 20). The questions in the 3 parts of the questionnaire were constructed based on the results of the co-design sessions in design stage 1. As we explained in design stage 1, mental health care professionals use eMH mainly because of its apparent benefits. To encourage this, the escape room should therefore highlight positive experiences with eMH in clinical practice, in line with the flow theory that states that a positive learning experience can be reached if the challenges meet the abilities of the users and if they can share this with colleagues [31]. To explore the extent to which the escape room was experienced positively, we formulated questions 1, 3, 9, 10, and 13. Furthermore, from design stage 1 and SDT, we learned that the educational content must align with real-life scenarios, making it easy to apply in practice [29]. Questions 6, 8, and 15 were therefore constructed to assess whether the storylines are recognizable and whether the training with eMH tools in these storylines connects to their daily practice. In addition, questions 2 and 5 were formulated to explore if the learning goals become clear, and questions 7 and 12 assessed whether the escape room helps to build confidence in using technology, with customizable elements tailored to specific needs. Finally, the game should enable multiplayer options for shared learning [31]. To assess whether the escape room was experienced that way, we added questions 4, 11, and 14. Together this led to the questionnaire, which had 20 targeted questions. The answers to the questions were subsequently used as a guide in discussion with the participants to gain a more in-depth understanding of the possibilities of an escape room as an environment for mental health care professionals to experiment and practice with eMH.

##### User Experiences

From the questionnaire and discussion, we learned that the participants in the field study sessions were enthusiastic about the workshop they participated in. They stated that the workshop is inspiring and creates a positive mindset toward eMH. They recognized the potential to remove barriers they had at first, showing that eMH is accessible and not always that difficult to use. The participants experienced significant fun in the escape room, making it feel less obligatory. This is expected to help people engage more easily. In addition, the alternation between playing and reflecting was mentioned as meaningful. In total, 2 (18%) of the 11 participants mentioned they were pleased that there was no explicit element of competition in the escape room. According to these participants, too much competition, for example, ranking who has solved the escape room the fastest, would distract the users from the storyline and the eMH possibilities they come across.

All but one participant expressed that they experienced the storylines in the escape room as realistic. One mental health care professional did not recognize the storylines in the escape room. Another aspect related to this was the “credibility” of the suggested effect of eMH in the escape room. Although most (9/11, 82%) of the participants could describe the use of eMH to certain benefits for mental health care, it should be carefully suggested in the escape room. However, most (9/11, 82%) participants experienced how they could practically apply eMH in therapeutic situations. For example, VR was experienced in the escape room, resulting in a discussion on how VR could be used for clients in specific treatment situations. The participants were able to connect their VR experience in the workshop to real-life situations and could see the added value of using eHealth. All kinds of eHealth suggestions were exchanged, which illustrates the effect of being given the possibility to explore and experiment with eHealth in this type of playful setting.

Participants wondered whether an escape room would appeal enough to therapists who were skeptical about eMH. The participants also mentioned that the current storylines and eMH tools may not be recognizable for all mental health care professionals. It was considered important to explore eMH tools that are also accessible in real life to be able to transfer knowledge from training to reality. In contrast, it is also mentioned that mental health care professionals with vast experience in eMH will find it more attractive to explore more experimental eMH tools.

##### Perceived Usability of an Escape Room for eMH Training

To explore the perceived usability of an escape room for skill enhancement in eMH, we asked the participants to indicate whether the workshop provided them with a more elaborate and precise picture of the possibilities of eMH (Table 2). The feedback on this issue varied among the participants. In total, 2 (18%) of the 11 mental health care professionals reported that they had not gained a better picture of the options of eMH in the escape room. This was because they already had a relatively good basic knowledge about eMH beforehand. The participants who were less experienced users of eMH reflected that they had a much better idea about the possibilities of eMH after participating in the escape room workshop. It was suggested that the current escape room prototype would benefit from further development to create more options to try additional eMH tools because, in the current prototype, not all mental health care professionals would find tools suitable for their therapeutic practice.

**Table 2:** Perceived usability of an escape room (ER) for e-mental health (eMH) training (questionnaire results).

Items	Score on a scale of 1-5, mean (SD)
Suitability of an ER to explore possibilities of eMH	4.7 (0.92)
Possibilities for collaboration in the ER	4.7 (0.47)
Clarity of the storylines	4.3 (0.92)
Clarity of learning goal in the ER	4.6 (0.67)
Balance educational content and fun	4.7 (0.68)
Usefulness for daily practice	3.8 (0.77)

The participants largely agreed on the suitability of an escape room concept for exploring eMH (average score of 4.7, SD 0.92). They perceived the learning goal in the workshop as evident (average score of 4.6, SD 0.67). They also experienced a good balance between the educational and the fun part in the escape room (average score of 4.7, SD 0.68). It was mentioned in the discussion that suitability is supported when the bridge between the escape room and everyday practice is explicitly discussed in the workshop as well as the steps that can be taken after completing the workshop and returning to the workplace. Follow-up training is essential because otherwise the acquired knowledge will not be secured. According to the participants, this escape room offers an excellent first experience with the possibilities of eMH; more than that, it is just another educational workshop, “It is more ‘getting to know eMH’ than ‘learning how to apply eMH.” It was also suggested that more focus on the client groups that people are treating would be helpful in the reflection part of the workshop. The participants advocated that involving professionals with similar client groups or treatment contexts would be more effective in stimulating the exchange of experiences with eMH and the transferability of the experiences in the escape room to daily practice.

In line with the findings about follow-up training, the participants elaborately reflect on how to apply and secure knowledge on eMH once the first experiences are gained in the escape room. While this workshop helps to talk about eMH, keeping the conversation going in everyday practice is equally important. According to the participants in our study, mental health care professionals are focused on the content of their treatment and support, and the knowledge gained in the eMH escape room might lose its impact. It was stated that the organization should provide continuous information about what is new in the field of eMH and should provide time to invest in exploring new tools. The concept of an escape room or a game offers a very persuasive opportunity to invest in keeping knowledge up to date and motivating people. All workshops suggested that this would be an excellent fit for a teambuilding activity.

##### Implementation Possibilities and Requirements

In addition to gathering experiences with the escape room workshop, we asked the mental health care professionals who participated to indicate what they deemed essential for successfully implementing it. The first thing mentioned was that professionals in mental health care should be confronted with eMH from various directions, that is, in an escape room, but also by clients, colleagues, and organizational policies aimed at being innovative, requiring innovative and digitally skilled employees. Incorporating an escape room in an introductory meeting might be possible when people are new to the organization.

Another issue that emerged from the workshops concerns the approachability of the escape room. It is experienced as convenient that the escape room is currently a mobile version, which does not necessarily require people to go somewhere and spend much time traveling. It would be even more convenient if the escape room could be played without an external facilitator so that participants could be more flexible in their time planning. However, this remains the main concern of the participants in the workshops: will mental health care professionals be provided with enough time to explore eMH in the future, and will their production targets be adjusted accordingly? In addition, it should be decided who will provide training services and materials in the organization. Finally, the participants stated that a strong vision by the policy makers on the use of eMH in the organization and the expectations regarding the future mental healthcare professional is indispensable to warrant a successful implementation of an escape room or any serious gaming concept for eMH training in mental health care.

## Discussion

### Principal Findings

In this research through design study, we investigated the perceived usability of an escape room for training mental health care professionals in using eMH. On the basis of the results in each stage of our research and the overall findings, we can conclude that an escape room concept might be suitable for this type of training. We subsequently discuss our findings by reflecting on the experiences with our escape room prototype and what this means in light of the purpose of our design project: developing new training methods for mental health care professionals, with the ultimate goal of contributing to increasing the adoption of eMH. We conclude with an overall view of the possibilities of game-based training in mental health care based on the results of this design study.

What we know from earlier research, which also emerged in this study, is that many mental health care professionals are not convinced of the benefits of eMH, resulting in dismissive attitudes and behavior [50,51]. The escape room was experienced as both fun and educational; however, most importantly, it induced a more positive stance toward eMH tools. While an open mindset is a prerequisite for change, the escape room may offer a potential concept as the first step to changing attitudes and behavior among mental health care professionals regarding eMH [36,40].

For the escape room to be accepted as a valuable training method by its end users, the storylines, game elements (puzzles and clues), and eMH tools in the escape room needed to be credible and recognizable for mental health care professionals. At the same time, it became clear that it is tough to design storylines that seamlessly match the practices of mental health care therapists. In one of the first versions of the prototype, there was too much focus on everyday life situations instead of mental health problems. This is crucial because the participants need a genuine eMH experience to link to their treatment context properly. This feedback was given in different iterations in the design process, which marks the difficulty of designing a good and representative storyline and the importance of involving multiple disciplines in a co-design team [46,47].

Moreover, it became clear that mental health care professionals are diverse in terms of their client population, type of mental health care services, and level of adoption in eMH. This requires a flexible and adaptable training concept to expand the reach of the escape room. Greater diversification regarding the storylines and available eMH tools in the escape room may be needed, along with various difficulty levels. The participants in the field study sessions indicated that the escape room in its current form might not be suitable for people who are already frequent users of eMH. This implies that the gains of the current escape room are probably highest for people who are relatively inexperienced eMH users, which applied to most mental health care professionals when we started the design process. Adding new, more innovative tools could, however, expand the reach of the escape room.

The participants welcomed the escape room as an engaging concept and noted that its design needs continuation and further development. In its current form, the escape room offers an environment for exploration and the first acquaintance with eMH rather than actual skill development in applying these tools. We believe that, whereas most mental health care professionals are—partly because of the coronavirus pandemic—familiar with standard eMH tools that are also used in everyday life (eg, videoconferencing and SMS text messaging), they remain unaware of the possibilities of more novel technologies, such as VR and biofeedback, and may be biased about its use in their daily clinical practice. Therefore, creating a different mindset remains an important foundation for further skill development in the future and increasing adoption in general. An extensive research base reports on various barriers that prevent accelerating the adoption of eMH [5,51,52]. This escape room addresses one of the critical barriers, a perceived lack of knowledge. However, issues such as compensating for the loss of nonverbal information, the lack of trust in technological systems, and the availability of time and resources to gain positive experiences in using eMH also need to be addressed.

When exploring ideas for further training for specific eMH tools, the question is whether an escape room would also be suitable for this particular type of training. Looking at escape rooms in health care, we find that escape rooms are most appropriate for exploration and awareness and less ideal for training-specific skills [23,24]. This was also why we chose an escape room concept in the first place; it suited the results of our user requirements analysis that revealed a specific need for exploring and enhancing knowledge rather than specific hands-on skills. Training these hands-on skills should be addressed for the next step, particularly because the participants in our study also indicated this need. Moreover, for both the possibility of exploration and specific skill training, the support in terms of time and resources provided by the organization and its management is crucial. The organization’s commitment to a digital transition and the necessity for mental health care professionals to be equipped with 21st-century skills must be at the forefront. Finally, follow-up research is required to test the escape room’s effectiveness regarding knowledge increase and skill enhancement.

### The Perceived Usability of Serious Gaming for eMH Training in Mental Health Care Based on Users’ Experiences

The results of our study show positive experiences with an escape room as a serious gaming concept for eMH training in mental health care. These positive experiences might be explained in terms of the SDT [29] and the flow theory [31]. By attempting to address the needs of competence and autonomy, the users may have had a positive experience. The flow of the escape room may have positively contributed to their motivation to enhance their knowledge of eMH and their general attitude toward using eMH in their clinical practice [30].

In the design of the escape room, we addressed several important game mechanics that we found to have influenced our users’ experiences, for example, an adequate level of complexity in the game [19,53,54]. The participants in our study emphasized that when a puzzle in the escape room takes too much time or is too difficult, it may lead to frustration and an adverse effect. The opposite of this also works negatively; when clues are too obvious, people may not feel they are taken seriously. In general, our escape room was experienced as having an appropriate level of difficulty, which led to a positive experience. We also paid much attention to the importance of the story or narrative in the escape room. When this narrative is too farfetched or not relatable to clinical practice, it loses strength; when people can identify with the story in the game, it may be much more effective than any other learning method [29-31]. In addition, the aspect of social interaction [30] was predominantly present in our escape room and experienced as one of the critical factors for its success.

### Strengths and Limitations

In this study, we particularly choose a research through design approach to develop and test an escape room as a serious gaming solution for eMH training among mental health care professionals. The strength of this approach is that it enabled us to use formative evaluations [55] between the stages of design to provide input for the next design stage. We strongly believe that without these intermediate assessments, we could not design a prototype suitable to the practice of mental health care providers. More importantly, the involvement of mental health care professionals in our co-design team ensured a prototype that matched the therapeutic practice we pursued to reflect in our escape room. However, there are some limitations. First of all, we used an inductive analysis method, which allowed us to gather any information deemed necessary for the development of the escape room but may have lacked the required structure to establish an evaluation of the effectiveness of the escape room, in terms of skill enhancement, and for other researchers to replicate the analyses. Analysis procedures that would allow an assessment of the escape room’s success could be to use a pre- and posttest quasi-experimental design for the evaluation [56], a controlled experiment with naturalistic and summative evaluations to assess whether the purpose of the game is indeed reached [57] or a heuristic evaluation using predefined rules of thumb to measure the usability of the escape room [58]. A second limitation of this study is that we included a small sample of mental health care professionals who all worked at the same mental health care organization. In other words, we did not include participants from a diverse mental health care setting other than the differentiation in mental health care contexts within an organization. In addition, we are aware that for projects like this, where participants are recruited through open invitation, people who are already enthusiastic about the topic, in this case, eMH, tend to be the ones who sign up for participation. In future research, it would be worthwhile to test the escape room or any other game-based training solution among mental health care professionals who are skeptical about eMH to see whether also, in this group, the effect of creating an open mindset through exploration and experience-based learning is achieved.

### Conclusions

In general, we can conclude that experience-based training, such as game-based training in an escape room, helps people experience what they are being trained to do or use. This experience may be much stronger than the experience of learning through traditional learning (eg, classroom, textbook, or e-learning). When training is more fun and engaging, this will increase the learning experience. Moreover, it increases the participants’ motivation and creates an open mindset. In our study, we learned that an escape room as a serious gaming concept stimulates the willingness to participate in training and be open to change. This openness to change is essential in increasing the use of eMH. However, it is important that there is a clear next step to this first exploration, providing professionals with the opportunity to thoroughly train their skills in specific eMH tools. Subsequently, it would be valuable to evaluate the effectiveness of game-based training in an escape room for enhancing particular skills. Furthermore, designing is a cooperation between different experts. The design process should be viewed from different angles and areas of expertise. The contribution of mental health care professionals was necessary to create a credible game in which both the storyline and set of integrated eMH tools can be transferred to their therapeutic practice. Finally, the success of a game-based training method, such as an escape room, depends on the commitment of the organization in which it is implemented. People need to feel that they have the time and support of their management to engage in training. This commitment is more than just telling them to “go and participate in training.” The whole organization must enhance specific knowledge based on common ground and a shared vision of what is needed regarding competencies and skills by mental health care professionals.

## Appendix

Multimedia Appendix 1 SQUIRE (Standards for Quality Improvement Reporting Excellence) checklist.

Multimedia Appendix 2 Results of the second design iteration in the development of an escape room for e-Mental health training.

Multimedia Appendix 3 Evaluation questions (English translation).

## Abbreviations

EER: educational escape room
eMH: e-Mental health
GGzE: Geestelijke Gezondheidszorg Eindhoven en de Kempen
SDT: self-determination theory
SMI: severe mental illness
VR: virtual reality

## Data Availability

The datasets generated and analyzed during this study are not publicly available due to agreements about confidentiality and exchange of data that were made with the respondents in the study. The consent given by the participants does not allow for public availability. The data can be made available from the corresponding author on reasonable request and with the consent of the participants in this study.

## References

[1] Andersson G, Cuijpers P, Carlbring P, Riper H, Hedman E (2014). Guided internet-based vs. face-to-face cognitive behavior therapy for psychiatric and somatic disorders: a systematic review and meta-analysis. World Psychiatry.

[2] de Grood C, Raissi A, Kwon Y, Santana M (2016). Adoption of e-health technology by physicians: a scoping review. J Multidisciplin Healthc.

[3] Glueckauf RL, Maheu MM, Drude KP, Wells BA, Wang Y, Gustafson DJ, Nelson EL (2018). Survey of psychologists’ telebehavioral health practices: technology use, ethical issues, and training needs. Prof Psychol Res Pract.

[4] Lal S, Adair CE (2014). E-mental health: a rapid review of the literature. Psychiatr Serv.

[5] Feijt MA, de Kort YA, Bongers IM, IJsselsteijn WA (2018). Perceived drivers and barriers to the adoption of eMental health by psychologists: the construction of the levels of adoption of eMental health model. J Med Internet Res.

[6] Vis C, Kleiboer A, Prior R, Bønes E, Cavallo M, Clark SA, Dozeman E, Ebert D, Etzelmueller A, Favaretto G, Zabala AF, Kolstrup N, Mancin S, Mathiassen K, Myrbakk VN, Mol M, Jimenez JP, Power K, van Schaik A, Wright C, Zanalda E, Pederson CD, Smit J, Riper H (2015). Implementing and up-scaling evidence-based eMental health in Europe: the study protocol for the MasterMind project. Internet Interv.

[7] Wozney L, Newton AS, Gehring ND, Bennett K, Huguet A, Hartling L, Dyson MP, McGrath P (2017). Implementation of eMental Health care: viewpoints from key informants from organizations and agencies with eHealth mandates. BMC Med Inform Decis Mak.

[8] Kilbourne AM (2012). E-health and the transformation of mental health care. Psychiatr Serv.

[9] Ross J, Stevenson F, Lau R, Murray E (2016). Factors that influence the implementation of e-health: a systematic review of systematic reviews (an update). Implement Sci.

[10] Chan CV, Kaufman DR (2011). A framework for characterizing eHealth literacy demands and barriers. J Med Internet Res.

[11] Norman C (2011). eHealth literacy 2.0: problems and opportunities with an evolving concept. J Med Internet Res.

[12] Schalken F (2013). Handboek Online Hulpverlening: Met Internet Zorg en Welzijn Verbeteren.

[13] Bierbooms JJ, van Haaren M, IJsselsteijn WA, de Kort YA, Feijt M, Bongers IM (2020). Integration of online treatment into the "new normal" in mental health care in post-COVID-19 times: exploratory qualitative study. JMIR Form Res.

[14] Crawford A, Gratzer D, Jovanovic M, Rodie D, Sockalingam S, Sunderji N, Teshima J, Thomas Z (2018). Building eHealth and telepsychiatry capabilities: three educational reports across the learning continuum. Acad Psychiatry.

[15] Echelard JF, Méthot F, Nguyen HA, Pomey MP (2020). Medical student training in eHealth: scoping review. JMIR Med Educ.

[16] Timman A, Caris JA (2022). Leren in een andere realiteit. Onderwijs en Gezondheidszorg.

[17] Connolly TM, Boyle EA, MacArthur E, Hainey T, Boyle JM (2012). A systematic literature review of empirical evidence on computer games and serious games. Comput Educ.

[18] Squire K, Jenkins H (2003). Harnessing the power of games in education. Insight.

[19] Wang R, DeMaria S Jr, Goldberg A, Katz D (2016). A systematic review of serious games in training health care professionals. Simul Healthc.

[20] Susi T, Johannesson M, Backlund P (2007). Serious games – an overview. Serious Games Cluster and Business Network Project.

[21] Makri A, Vlachopoulos D, Martina RA (2021). Digital escape rooms as innovative pedagogical tools in education: a systematic literature review. Sustainability.

[22] Ritterfeld U, Cody M, Vorderer P (2009). Serious Games: Mechanisms and Effects.

[23] Adams V, Burger S, Crawford K, Setter R (2018). Can you escape? Creating an escape room to facilitate active learning. J Nurses Prof Dev.

[24] Mullen M, Seiler SO (2019). Escaping the confines of traditional instruction: the use of the escape room in a transition to practice program. J Nurses Prof Dev.

[25] Veldkamp A, van de Grint L, Knippels MC, van Joolingen WR (2020). Escape education: a systematic review on escape rooms in education. Educ Res Rev.

[26] Kolb DA, Boyatzis RE, Mainemelis C, Sternberg RJ, Zhang LF (2014). Experiential learning theory: previous research and new directions. Perspectives on Thinking, Learning, and Cognitive Styles.

[27] Prensky M (2001). Fun, play and games: what makes games engaging. Digital Game-Based Learning.

[28] Grande-de-Prado M, García-Martín S, Baelo R, Abella-García V (2020). Edu-escape rooms. Encyclopedia.

[29] Deci EL, Ryan RM (2004). Handbook of Self-Determination Research.

[30] Vorderobermeier A, Abel J, Sailer M (2024). Theoretical foundations and approaches in research on educational escape rooms: a systematic review. Educ Res Rev.

[31] Csikszent M (1991). Flow: The Psychology of Optimal Experience.

[32] Deen M (2015). G.A.M.E., Games autonomy motivation & education: how autonomy-supportive game design may improve motivation to learn. Technische Universiteit Eindhoven.

[33] Okuda Y, Bryson EO, DeMaria S Jr, Jacobson L, Quinones J, Shen B, Levine AI (2009). The utility of simulation in medical education: what is the evidence?. Mt Sinai J Med.

[34] Reeves B, Read JL (2009). Total Engagement: How Games and Virtual Worlds Are Changing the Way People Work and Businesses Compete.

[35] Rodriguez-Ferrer JM, Manzano-León A, Cangas AJ, Aguilar-Parra JM (2022). A web-based escape room to raise awareness about severe mental illness among university students: randomized controlled trial. JMIR Serious Games.

[36] Aragon CJ (2023). Determining the effectiveness of an escape room in a mental health nursing course: a quantitative study. Northcentral University.

[37] Petkari E, Calvo A (2024). Escaping psychodiagnostics: implementation of a Digital Educational Escape Room with future mental health professionals. Interact Learn Environ.

[38] Bierbooms J, Feijt M, IJsselsteijn W, Sluis-Thiescheffer W, Weijmans M, van der Burg E, Bongers I (2020). Serious games for professional skills: the design of an escape room to explore the possibilities of eMental health. Proceedings of the Companion Publication of the 2020 ACM Designing Interactive Systems Conference.

[39] Bierbooms J, Feijt MA, IJsselsteijn WA, Bongers IM (2022). Design of a game-based training environment to enhance mental health care professionals' skills in using e-mental health: multiple methods user requirements analysis. JMIR Serious Games.

[40] Pan R, Lo H, Neustaedter C (2017). Collaboration, awareness, and communication in real-life escape rooms. Proceedings of the 2017 Conference on Designing Interactive Systems.

[41] Djaouti D, Alvarez J, Jessel JP (2011). Classifying serious games: the G/P/S model. Handbook of Research on Improving Learning and Motivation through Educational Games: Multidisciplinary Approaches.

[42] Stappers PJ, Giaccardi E, Soegaard M, Friis-Dam R (2014). Research through design. The Encyclopedia of Human-Computer Interaction.

[43] Zimmerman J, Forlizzi J, Evenson S (2007). Research through design as a method for interaction design research in HCI. Proceedings of the SIGCHI Conference on Human Factors in Computing Systems.

[44] Jaarverslag GGzE 2020: werken aan mentale vooruitgang. GGzE.

[45] Donetto S, Pierri P, Tsianakas V, Robert G (2015). Experience-based co-design and healthcare improvement: realizing participatory design in the public sector. Des J.

[46] Jessen S, Mirkovic J, Ruland CM (2018). Creating gameful design in mHealth: a participatory co-design approach. JMIR Mhealth Uhealth.

[47] Robert G, Ziebland S, Coulter A, Calabrese JD, Locock L (2013). Participatory action research: using experience-based co-design to improve the quality of healthcare services. Understanding and Using Health Experiences: Improving Patient Care.

[48] Boyatzis RE (1998). Transforming Qualitative Information: Thematic Analysis and Code Development.

[49] Ogrinc G, Davies L, Goodman D, Batalden P, Davidoff F, Stevens D (2016). SQUIRE 2.0 (Standards for QUality Improvement Reporting Excellence): revised publication guidelines from a detailed consensus process. BMJ Qual Saf.

[50] Connolly SL, Miller CJ, Lindsay JA, Bauer MS (2020). A systematic review of providers' attitudes toward telemental health via videoconferencing. Clin Psychol (New York).

[51] Davies F, Shepherd HL, Beatty L, Clark B, Butow P, Shaw J (2020). Implementing web-based therapy in routine mental health care: systematic review of health professionals' perspectives. J Med Internet Res.

[52] Stoll J, Müller JA, Trachsel M (2019). Ethical issues in online psychotherapy: a narrative review. Front Psychiatry.

[53] Ijaz A, Khan MY, Ali SM, Qadir J, Kamel Boulos MN (2019). Serious games for healthcare professional training: a systematic review. Eur J Biomed Inform.

[54] Rooney P (2012). Creating serious games at third level: evaluating the implications of an in-house approach. Proceedings of the European Conference on Game Based Learning.

[55] van Gemert-Pijnen JE, Nijland N, van Limburg M, Ossebaard HC, Kelders SM, Eysenbach G, Seydel ER (2011). A holistic framework to improve the uptake and impact of eHealth technologies. J Med Internet Res.

[56] Moizer J, Lean J, Dell’Aquila E, Walsh P, Keary A, O'Byrne D, Di Ferdinando A, Miglino O, Friedrich R, Asperges R, Sica LS (2019). An approach to evaluating the user experience of serious games. Comput Educ.

[57] Pistono AM, dos Santos AM, Baptista RJ, Mamede HS (2024). Framework for adaptive serious games. Comput Appl Eng Educ.

[58] Karavidas L, Apostolidis H, Tsiatsos T (2022). Usability evaluation of an adaptive serious game prototype based on affective feedback. Information.

