# Supplementary material for: Co-Design of an Escape Room for e-Mental Health Training of Mental Health Care Professionals: Research Through Design Study
Source: JMIR Form Res. 2025 Jan 7;9:e58650. doi: 10.2196/58650 (PMC11751646; doi:10.2196/58650)
Supplement: Multimedia Appendix 2 [file formative_v9i1e58650_app2.docx]

Table 2. Results of the second design iteration in the development of an escape room for eMH training.

| Iteration | Feedback | Adaptations |
| --- | --- | --- |
| 1: try-out version | - Storylines must reflect truly relatable situations. Otherwise, eMH tools cannot be linked to actual solutions. - Transferability to practice can still be improved. - Focus on solving the puzzles should be balanced, and eMH experience should be central. - More variation is needed to make the escape room interesting for a broad user group. - A clear conclusion of the game is lacking. | - Addition of complexity to the storylines. - Addition of a storyline with other mental health problems. - Addition of eMH tools. - Better connections between eMH tools and solving the puzzles. - Adding a clear and notable ‘end-point.’ |
| 2: online version | - Multiple technical hassles impeded the flow of the game. - The online version lacks the important possibility of playing with colleagues and exchanging ideas. | - Technical hassles that were important for the physical version of the escape room were solved. - The final choice to pursue a physical escape room (at this point) was made. |
| 3: final prototype | - A few technical details should be improved, e.g., being unable to go on with the game without using an eMH tool. - Language should be suitable for the target group. - Small adaptations regarding the storylines would further improve the escape room. - Introduction and reflection need to be developed. | - Minor adaptations regarding storylines, game flow, and puzzles. - Addition of introduction and reflection to the game. |
