# Supplementary material for: Co-Design of an Escape Room for e-Mental Health Training of Mental Health Care Professionals: Research Through Design Study
Source: JMIR Form Res. 2025 Jan 7;9:e58650. doi: 10.2196/58650 (PMC11751646; doi:10.2196/58650)
Supplement: Multimedia Appendix 3 [file formative_v9i1e58650_app3.pdf]

Evaluation questions Escape Room

We would like to ask you to evaluate today's workshop. You can answer the different questions on a scale from 1 to 5. At the end, we ask you to provide your strengths and suggestions.

Overall experience

We are curious to know your overall experience after participating in the workshop.

1. How do you assess this experience?

Very Poor				Very good
1	2	3	4	5

Introduction

In the introduction we elaborated on the project and the purpose of the reseach.

2. How clear was the story in the introduction?

Very unclear				Very clear
1	2	3	4	5

3. How did you experience the duration of the introduction?

Too short		Good		Too long
1	2	3	4	5

4. How did you experience the possibility for interaction during the introduction?

Very Poor				Very good
1	2	3	4	5

Playing the Escape Room

You met Victor and Anouk, after which you worked with one of them in the Escape Room.

5. To what extent was the learning goal clear to you in the Escape Room?

GGzE

Very unclear Very clear

1 2 3 4 5

6. Did you get a better view on the possibilities of eMental Health by this workshop?

Very little Very much

1 2 3 4 5

7. How suitable do you think an escape room is to get acquainted with eMH?

Very little Very much

1 2 3 4 5

8. Were the storylines in the Escape Room realistic?

Very little Very much

1 2 3 4 5

9. How did you experience the duration of playing the Escape Room?

Too short Good Too long

1 2 3 4 5

10. How did you experience the balance between education and fun in the Escape Room?

Very Poor Very good

1 2 3 4 5

11. To what extent did you experience playing together?

Very little Very much

1 2 3 4 5

Reflection

After playing the game, we reflected on the Escape Room together.

12. Were the reflection questions clear?

Very unclear Very clear

1 2 3 4 5

13. How did you experience the duration of the reflection?

Too short Good Too long

1 2 3 4 5

14. How did you experience the possibility for interaction during the introduction?

Very Poor Very good

1 2 3 4 5

15. How do you assess the content of the reflection questions?

Very Poor Very good

1 2 3 4 5

Usability

Finally, we would like to ask you some questions about how you experienced the usability of the escape room workshop for your daily practice.

16. How did you experience the balance between the time you invested and the gains you received from participating in the workshop?

Very Poor Very good

1 2 3 4 5

17.

18. To what extent do you think you can use the gains from the workshop for you daily practice?

Very little Very much

1 2 3 4 5

19. Which organizational department should facilitate this escape room (multiple answers allowed)?

GGzE

- ☐ Board / management
- ☐ Team learning and development
- ☐ eHealth support team
- ☐ Team Policy & Strategy
- ☐ HRM
- ☐ In company training facilities
- ☐ ICT
- ☐ Other, …

…………………………………………………………………………….

20. Can you provide one strength and one suggestion for further development and implementation of the escape room?

Strength:……

Suggestion:……..
